# Sex‐specific newborn screening for X‐linked adrenoleukodystrophy

**DOI:** 10.1002/jimd.12571

**Published:** 2022-10-26

**Authors:** Monique Albersen, Samantha L. van der Beek, Inge M. E. Dijkstra, Mariëlle Alders, Rinse W. Barendsen, Jet Bliek, Anita Boelen, Merel S. Ebberink, Sacha Ferdinandusse, Susan M. I. Goorden, Annemieke C. Heijboer, Mandy Jansen, Yorrick R. J. Jaspers, Ingrid Metgod, Gajja S. Salomons, Frédéric M. Vaz, Rendelien K. Verschoof‐Puite, Wouter F. Visser, Eugènie Dekkers, Marc Engelen, Stephan Kemp

**Affiliations:** ^1^ Endocrine Laboratory, Department of Clinical Chemistry Amsterdam UMC location University of Amsterdam, Amsterdam Gastroenterology Endocrinology Metabolism Amsterdam The Netherlands; ^2^ Reference Laboratory for Neonatal Screening, Center for Health Protection National Institute for Public Health and the Environment (RIVM) Bilthoven The Netherlands; ^3^ Laboratory Genetic Metabolic Diseases, Department of Clinical Chemistry Amsterdam UMC Location University of Amsterdam, Amsterdam Neuroscience, Amsterdam Gastroenterology Endocrinology Metabolism Amsterdam The Netherlands; ^4^ Department of Human Genetics Amsterdam UMC location University of Amsterdam, Amsterdam Reproduction & Development Amsterdam The Netherlands; ^5^ Endocrine Laboratory, Department of Clinical Chemistry Amsterdam UMC location Vrije Universiteit Amsterdam, Amsterdam Gastroenterology Endocrinology Metabolism Amsterdam The Netherlands; ^6^ Department for Vaccine Supply and Prevention Programs National Institute for Public Health and the Environment (RIVM) Bilthoven The Netherlands; ^7^ Department of Pediatric Neurology Amsterdam UMC location University of Amsterdam, Amsterdam Leukodystrophy Center, Emma Children's Hospital, Amsterdam Neuroscience Amsterdam The Netherlands; ^8^ Center for Population Screening National Institute for Public Health and the Environment (RIVM) Bilthoven The Netherlands

**Keywords:** *ABCD1*, adrenoleukodystrophy, C26:0‐LPC, dried bloodspots, heel prick, newborn screening, sex‐specific, X‐chromosome

## Abstract

Males with X‐linked adrenoleukodystrophy (ALD) are at high risk for developing adrenal insufficiency and/or progressive leukodystrophy (cerebral ALD) at an early age. Pathogenic variants in *ABCD1* result in elevated levels of very long‐chain fatty acids (VLCFA), including C26:0‐lysophosphatidylcholine (C26:0‐LPC). Newborn screening for ALD enables prospective monitoring and timely therapeutic intervention, thereby preventing irreversible damage and saving lives. The Dutch Health Council recommended to screen only male newborns for ALD without identifying untreatable conditions associated with elevated C26:0‐LPC, like Zellweger spectrum disorders and single peroxisomal enzyme defects. Here, we present the results of the SCAN (Screening for ALD in the Netherlands) study which is the first sex‐specific newborn screening program worldwide. Males with ALD are identified based on elevated C26:0‐LPC levels, the presence of one X‐chromosome and a variant in *ABCD1*, in heel prick dried bloodspots. Screening of 71 208 newborns resulted in the identification of four boys with ALD who, following referral to the pediatric neurologist and confirmation of the diagnosis, enrolled in a long‐term follow‐up program. The results of this pilot show the feasibility of employing a boys‐only screening algorithm that identifies males with ALD without identifying untreatable conditions. This approach will be of interest to countries that are considering ALD newborn screening but are reluctant to identify girls with ALD because for girls there is no direct health benefit. We also analyzed whether gestational age, sex, birth weight and age at heel prick blood sampling affect C26:0‐LPC concentrations and demonstrate that these covariates have a minimal effect.

## Graphical abstract

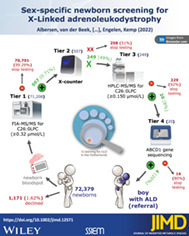

## 1 INTRODUCTION

X‐linked adrenoleukodystrophy (ALD; OMIM: 300100) is caused by a deficiency of the ABC half‐transporter encoded by the *ABCD1* gene.^1^ Pathogenic variants in the ABCD1 protein impair the import of very long‐chain fatty acids (VLCFA) into peroxisomes for degradation,^2^ causing accumulation of VLCFA in plasma and tissues, including adrenal glands, spinal cord and brain.^3^ ALD is characterized by an unpredictable clinical spectrum that includes primary adrenal insufficiency, progressive myeloneuropathy and cerebral inflammatory disease (cerebral ALD).^4^ The clinical outcome of an individual patient cannot be predicted due to the lack of a genotype–phenotype correlation and the absence of predictive molecular markers, and even relatives sharing identical *ABCD1* pathogenic variants can have different clinical outcomes.^5^, ^6^, ^7^

Patients with ALD are asymptomatic at birth. In males, the lifetime prevalence of developing adrenal insufficiency is 80%, but 50% of boys will be affected in the first decade of life.^8^, ^9^ Adrenal insufficiency can be treated effectively with corticosteroid replacement therapy, but the diagnosis is often delayed or missed because signs and symptoms are nonspecific.^10^ Untreated adrenal failure may lead to significant morbidity.^11^ Male patients are at risk of developing cerebral ALD, with the highest incidence (30%–40%) in boys between 3 and 12 years of age.^9^ Hematopoietic stem cell transplantation halts progression of cerebral ALD if initiated at the earliest stage of the disease, when neurological examination is normal and the lesion load on brain MRI is low.^12^, ^13^ Adrenal insufficiency and/or cerebral ALD are exceedingly rare in women with ABCD1 deficiency. Some isolated cases that were shown to have completely skewed X‐inactivation toward the mutated *ABCD1* allele have been reported.^14^, ^15^ In adulthood, virtually all male patients and >80% of women with ALD develop a chronic and slowly progressive myeloneuropathy for which no disease‐modifying therapy is available.^16^, ^17^, ^18^

Newborn screening for ALD enables prospective monitoring and timely therapeutic intervention, thereby preventing irreversible damage and saving lives. ALD newborn screening started in December 2013 in New York State.^19^, ^20^ At present, over 25 US states have added ALD to their screening program.^19^, ^21^, ^22^, ^23^, ^24^, ^25^, ^26^, ^27^ Outside the US, Taiwan initiated ALD newborn screening in November 2016,^28^ and a pilot is ongoing in Japan.^29^

In 2015, the Health Council of the Netherlands advised the Ministry of Health, Welfare and Sport (VWS) to expand the Dutch newborn screening panel with 14 conditions, including ALD.^30^, ^31^ These conditions are added in a phased manner with 1–3 diseases per year. The Dutch Health Council adheres to the Wilson and Jungner criteria,^32^ and recommended to only screen male newborns for ALD, because boys are at high risk of developing adrenal insufficiency and/or cerebral ALD and therefore will have direct benefit from newborn screening. The Health Council recommended that the identification of girls with ALD or boys and girls with other untreatable conditions associated with elevated C26:0‐LPC levels, like Zellweger spectrum disorders and single peroxisomal enzyme defects, is not warranted at this time. The novelty of sex‐specific newborn screening and the lack of an example for a boys‐only screening algorithm required a pilot study before ALD can be included in the nationwide screening program. Commissioned by the Ministry of Health, Welfare and Sport, the ALD group of the Amsterdam UMC, together with the National Institute for Public Health and the Environment (RIVM) designed this pilot study,^31^ of which the acronym became the SCAN study (Screening for ALD in the Netherlands).

ALD newborn screening is based on the analysis of C26:0‐lysophosphatidylcholine (C26:0‐LPC) in newborn heel prick dried blood spots. C26:0‐LPC concentrations are elevated in all ALD males and females, both in newborns and adult patients.^33^, ^34^ Most ALD newborn screening programs follow the 3‐tier algorithm developed by New York State.^20^ In Tier 1, C26:0‐LPC is quantified in a high‐throughput flow injection analysis/tandem mass spectrometric (FIA–MS/MS) method. Due to the presence of an unknown isobaric interferent present in blood, samples that are above the cut‐off are re‐analyzed in Tier 2 using the more sensitive and specific high‐performance liquid chromatography (HPLC)‐MS/MS method. Finally, Tier 3 consists of sequencing the *ABCD1* gene.^35^ The Dutch ALD screening algorithm is based on this 3‐tier algorithm, but with the addition of an extra tier we developed previously (the X‐counter) that enables identification of newborns with one X‐chromosome.^31^ The X‐counter is integrated as second tier in our 4‐tier screening algorithm.

Here, we present the results of the 1‐year SCAN study carried out in a part of the Netherlands. The main objectives of this pilot were to test our boys‐only screening algorithm in a newborn screening program and to identify males with ALD without identifying untreatable conditions also characterized by increased C26:0‐LPC concentrations. Additionally, the effect of gestational age, sex, birth weight and age at heel prick blood sampling on C26:0‐LPC concentrations was investigated.

## 2 MATERIALS AND METHODS

### 2.1 Subjects, informed consent, and blood sampling

The SCAN study protocol was reviewed by the Institutional Review Board of the Amsterdam UMC (METC W18_433 # 19.017), and deemed exempt because the Medical Research Involving Human Subjects Act (WMO) does not apply. Dried blood spot (Guthrie) cards that were sampled from January 1st to December 31st 2021 from newborns (boys and girls) in the participating Dutch provinces (Noord‐Holland, Utrecht, Flevoland and Gelderland) were included. Parents of boys and girls were asked to participate in the SCAN study. To enable parents to provide or deny informed consent, an information brochure (in Dutch and English) and a website (www.scanstudie.nl; with translations of the information brochure in Polish, Turkish and Arabic) were developed. In the third trimester of pregnancy, the obstetric care provider informed expecting parents about newborn screening and the SCAN study, and provided them with the information brochure. At birth registration at the municipality, parents also received the information brochure. Newborn heel prick blood collection is performed as soon as possible between 72 and 168 h after birth. Before performing the heel prick, informed consent for participation in the SCAN study was requested. If parents decided not to participate, this was marked on the Guthrie card and the C26:0‐LPC result was not registered. After completion of the pilot, and with permission of the relevant committee of the Department for Vaccine Supply and Prevention Programs (DVP) of the RIVM, anonymized information regarding gestational age, sex, birth weight and age at heel prick blood sampling was obtained for covariate analysis.

### 2.2 Tier 1: analysis of C26:0‐LPC by flow injection analysis/tandem mass spectrometry (FIA–MS/MS)

Two of the five Dutch newborn screening laboratories participated in the pilot.^31^ The analysis of C26:0‐LPC is performed using the NeoBase™ 2 non‐derivatized MSMS kit, that includes the internal standard for C26:0‐LPC, d4‐C26:0‐LPC (PerkinElmer), on a Waters Xevo TQD according to the manufacturer's instructions. Single 3.2 mm discs were punched from the Guthrie cards into 96‐well plates, after which 125 μl of the extraction working solution (EWS) was added to each well. The microplate was covered with an adhesive microplate cover and shaken for 30 min with 650–750 rpm at 45°C. The microplate cover was removed, 100 μl of the extraction was transferred to a new microplate and covered again with an adhesive microplate cover before being analyzed.

### 2.3 Tier 2: determination of the number of X‐chromosomes (X‐counter)

The procedure used to determine the number of X‐chromosomes present in Tier 1 screen positive dried blood spots has been described in detail before.^31^ In short, the number of X‐chromosomes is determined by assessing the ratio of three different non‐polymorphic markers that are present both on the X‐chromosome and an autosomal chromosome (chromosome 2, 3, and 7, respectively). A 1:1 ratio indicates a female and a 1:2 ratio indicates a male. To prevent the identification of unsolicited findings, such as a micro deletion that causes one marker to fail, the test is considered successful when the two remaining markers show the same result.

### 2.4 Tier 3: analysis of C26:0‐LPC by high‐performance liquid chromatography (HPLC–MS/MS)

A single 3.2 mm punch from a dried blood spot was extracted with 10 μl internal standard solution containing 1 μM d4‐C26:0‐LPC (Avanti Polar Lipids) in 0.25 ml of methanol, by ultrasonication in an ultrasonic bath (Branson 3510) for 5 min at room temperature. The extract was transferred to a 1.5 ml crimp neck vial and methanol was evaporated under a constant stream of nitrogen at 40°C. The residue was dissolved in 50 μl of methanol, transferred to a sample vial, and capped. Analysis was performed using an ACQUITY HPLC system (Waters Corp., Milford, MA, USA) consisting of a binary solvent manager, vacuum degasser, column heater and a sample manager. Two μL of the extract was injected onto a Kinetex C8 column at 50°C (50 × 2.1 mm, 2.6 μm particle diameter; Phenomenex, Torrance, CA). Metabolites were separated using a linear gradient between solution A (0.1% formic acid in H_2_O) and solution B (0.1% formic acid in methanol). The HPLC run was 10 min at a flow rate of 0.4 ml/min. All gradient steps were linear and as follows: at *T* = 0 min: 64% B, toward *T* = 6 min: 100% B; *T* = 6–11 min 100% B isocratic, and *T* = 11–12 min back to 64% B. Detection was done using a Quattro Premier XE mass spectrometer (Waters, Milford, MA) was used in the positive electrospray ionization mode. The spray voltage used was 3.5 kV. Nitrogen was used as desolvation gas (10.83 L/min) as well as cone gas (0.83 L/min). Desolvation and source temperatures were 350°C and 150°C, respectively. Argon was used as collision gas (2.5 × 10 e‐3 mbar). For C26:0‐LPC, the following MRMs were used: C26:0‐LPC (636.50 > 104.10) and d4‐C26:0‐LPC (640.50 > 104.10) with a dwell time of 0.165 s, a cone voltage of 20 V and a collision energy of 29 eV. C26:0‐LPC concentrations were calculated with Masslynx 4.0 software using the Quanlynx module.

### 2.5 Tier 4: *ABCD1* gene‐sequencing

*ABCD1* gene variant analysis was performed according to the protocol described by Boehm and coworkers.^35^ Variants were annotated according to the ACMG guidelines using the “Alamut™ Visual Plus” software package (Sophia Genetics) with transcript NM_000033.3 on GRCh37 (hg19) as the reference sequence.

### 2.6 Functional testing of VLCFA metabolism in skin fibroblasts

The tests to assess the effect of a variant of uncertain significance (VUS) identified in *ABCD1* on VLCFA metabolism in skin fibroblasts, that is, C26:0‐LPC and C26:0 levels, the C26:0/C22:0 ratio, peroxisomal beta‐oxidation and de novo VLCFA synthesis, have been described in detail by van de Stadt and coworkers.^36^ Of note, this functional testing of VLCFA metabolism is not part of the newborn screening algorithm. It is performed as part of the follow‐up protocol after referral to the pediatric neurologist.

## 3 RESULTS

### 3.1 Outcomes of a 1‐year pilot

**FIGURE 1:**
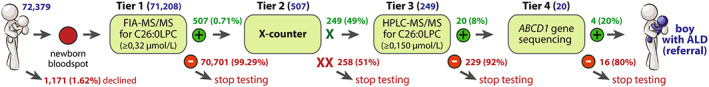
The Dutch 4‐tier ALD newborn screening algorithm. In the Dutch 4‐tier ALD newborn screening algorithm, males with ALD are identified based on elevated C26:0‐LPC concentrations, the presence of one X‐chromosome and a variant in *ABCD1*, in heel prick dried bloodspots. The first tier consists of quantification of C26:0‐LPC using a high‐throughput flow injection analysis/tandem mass spectrometric (FIA–MS/MS) method. Samples that are above the cut‐off are screened in Tier 2 for the presence of one X‐chromosome. Next, C26:0‐LPC is re‐analyzed in Tier 3 using the more sensitive and specific high‐performance liquid chromatography (HPLC)‐MS/MS method. If C26:0‐LPC is above the cut‐off the *ABCD1* gene is sequenced in Tier 4. Numbers in black and in parentheses indicate the number of samples analyzed in each tier. Numbers and percentages indicate the number and percentage of screen positive (green) and screen negative (red) samples in each tier.

During the pilot 72 379 children were offered ALD screening (Figure 1). Parents of 1171 (1.62%) newborns declined to participate in the SCAN study. The mean C26:0‐LPC concentration in heel prick dried blood spots of the resulting 71 208 cards that were screened by FIA–MS/MS in Tier 1 was 0.16 ± 0.05 μmol/L (mean ± SD; range 0.01–1.11). For 507 (0.71%) of these newborns the C26:0‐LPC concentration was above the cut‐off level (≥0.32 μmol/L), and they were referred to Tier 2 for sex determination (X‐counter). Analysis of the number of X‐chromosomes for these newborns resulted in 249 (49.1%) boys and 258 (50.9%) girls. The mean C26:0‐LPC concentration in dried blood spots of the 249 cards that were subsequently screened by HPLC–MS/MS in Tier 3 was 0.119 ± 0.171 μmol/L (mean ± SD; range 0.043–1.809). Twenty newborns had a C26:0‐LPC concentration above the cut‐off level (≥0.150 μmol/L), so the *ABCD1* gene was analyzed in Tier 4. In four newborns an *ABCD1* variant was identified (cases XY12, XY16, XY17, and XY18; Table 1). These newborns were referred to the pediatric neurologist at the Amsterdam Leukodystrophy Center of the Amsterdam UMC. Two male newborns (cases XY19 and XY20) had C26:0‐LPC levels that were higher than those measured in boys earlier diagnosed with ALD,^34^ but no variant in *ABCD1* was identified. It is possible that among the 16 boys without an *ABCD1* variant boys with a Zellweger spectrum disorder or a single peroxisomal enzyme defect were present. Following the recommendation of the Health Council of the Netherlands further genetic testing in these boys was not pursued.

**TABLE 1:** Newborns detected in the Dutch ALD newborn screening pilot

Case	C26:0‐LPC (FIA)^a^	C26:0‐LPC (HPLC)^a^	*ABCD1* variant	Variant interpretation	Cataloged in *ABCD1* variant database?	Inheritance	Family history
XY01	0.32	0.172	None	–	–	–	–
XY02	0.32	0.232	None	–	–	–	–
XY03	0.32	0.150	None	–	–	–	–
XY04	0.33	0.169	None	–	–	–	–
XY05	0.33	0.154	None	–	–	–	–
XY06	0.33	0.176	None	–	–	–	–
XY07	0.33	0.222	None	–	–	–	–
XY08	0.33	0.166	None	–	–	–	–
XY09	0.33	0.205	None	–	–	–	–
XY10	0.33	0.206	None	–	–	–	–
XY11	0.33	0.153	None	–	–	–	–
XY12	0.33	0.242	c.1253G>A (p.Arg418Gln)	Likely pathogenic^b^	Yes	Maternal	No
XY13	0.34	0.220	None	–	–	–	–
XY14	0.35	0.200	None	–	–	–	–
XY15	0.37	0.159	None	–	–	–	–
XY16	0.39	0.326	c.574A>G (p.Asn192Asp)	Likely pathogenic^b^	No	Maternal	No
XY17	0.64	0.909	c.1850G>A (p.Arg617His)	Pathogenic	Yes	Maternal	No
XY18	0.68	0.840	c.1781‐1G>C (Splice site)	Likely pathogenic^b^	No	De novo	No
XY19	1.07	1.80	None	–	–	–	–
XY20	1.11	1.81	None	–	–	–	–

*Note*: “–” not applicable.

^a^ C26:0‐LPC concentration determined by FIA–MS/MS (FIA) and HPLC–MS/MS (HPLC) in μmol/L.

b These variants were initially classified as VUS, but following functional testing in fibroblasts they were reclassified as likely pathogenic.

### 3.2 Referral and confirmatory testing

In case of a positive screening result (*ABCD1* variant in Tier 4) the medical advisor of the RIVM contacts the child's family physician and the child is referred to the Amsterdam Leukodystrophy Center (Amsterdam UMC). In all four cases, the time between the family being notified of a positive screening result for ALD by their family physician and the first consultation with the pediatric neurologist was 1 day. At this first visit, the pediatric neurologist provided the family detailed information on the disease as well as the recommended follow‐up and a blood sample was taken to confirm the diagnosis. In all four newborns the diagnosis ALD was confirmed. A second visit was scheduled at the age of 6–8 weeks. At this second visit, adrenal function was tested, genetic consultation and extended family screening were offered and initiated, and all four boys enrolled in a long‐term follow‐up program with repeat visits every 6 months as published in detail earlier.^31^ Maternal inheritance of the *ABCD1* variant was confirmed in three cases and one boy carried a de novo *ABCD1* variant (Table 1). None of the families reported a family history of ALD, but extended family screening to identify at‐risk males is ongoing.

Initially, three variants were classified as VUS (Table 1). A skin biopsy was taken for functional testing of VLCFA metabolism in fibroblasts with the aim to (re)classify these variants as likely benign or likely pathogenic.^36^ Compared to control fibroblasts, all three cell lines showed abnormal VLCFA metabolism: C26:0‐LPC and total C26:0 levels were elevated with an increased C26:0/C22:0 ratio, d3‐C26:0 synthesis was increased and d3‐C22:0 beta‐oxidation was reduced (Table 2). Therefore, all three variants were reclassified as likely pathogenic, yielding a positive predictive value (PPV) of 100% for the ALD pilot screening algorithm (4/4).

**TABLE 2:** Functional analyses in fibroblasts of new variants identified in *ABCD1*

Test	Reference range^a^	p.Asn192Asp (XY16)	p.Arg418Gln (XY12)	c.1781‐1G>C (splice site) (XY18)	ALD range^a^
C26:0‐LPC (pmol/mg protein)	2–14	38	25	30	>20
d3‐C26:0 synthesis (nmol/mg protein)	0.15–0.51	2.51	1.57	3.27	>1.00
d3‐C22:0 beta‐oxidation^b^	>57%	12%	25%	11%	12%–26%
C26:0 (nmol/mg protein)	0.18–0.39	0.82	0.87	1.11	>0.81
C26:0/C22:0 ratio	0.03–0.10	0.34	0.33	0.60	>0.21
Classification		Likely pathogenic	Likely pathogenic	Likely pathogenic	

^a^ Control and ALD ranges for these tests were established earlier using 36 cell lines derived from male ALD patients and 26 cell lines derived from control subjects and described in detail by van de Stadt et al.^36^

b d3‐C22:0 beta‐oxidation is expressed as a percentage of the mean activity in control cells analyzed in the same experiment.

### 3.3 Analytical turnaround times

Analytical turnaround times are important, because in the Netherlands parents expect a letter containing the newborn screening results no later than 35 days after the heel prick is performed. The analytical turnaround times during the SCAN study pilot were 7 ± 3 days for Tier 1 – Tier 2 (*n* = 507), 12 ± 4 days for Tier 1 – Tier 3 (*n* = 249) and 21 ± 9 days for Tier 1 – Tier 4 (*n* = 20), thereby not exceeding this time limit of 35 days.

### 3.4 Evaluation of the X‐counter

To enable sex‐specific newborn screening, we developed and validated the X‐counter in a laboratory setting.^31^ To evaluate X‐chromosome analysis during the pilot, we compared results obtained with the X‐counter (*n* = 507) with the sex information provided on the Guthrie card. In 505 out of 507 (99.6%) analyses the results of the X‐counter agreed with the sex information provided on the Guthrie card. Analysis of the two discrepancies revealed that one case was a girl that was marked “boy” on the Guthrie card. The X‐counter prevented screening of this girl for ALD. The second case was a boy, as determined by the X‐counter, that was marked “girl” on the Guthrie card. Without the X‐counter, this boy would not have been screened for ALD. For both cases, re‐analysis of an additional dried blood spot punch of the original Guthrie card excluded sample mix‐up.

### 3.5 Comparison of C26:0‐LPC levels between FIA–MS/MS and HPLC–MS/MS

**FIGURE 2:**
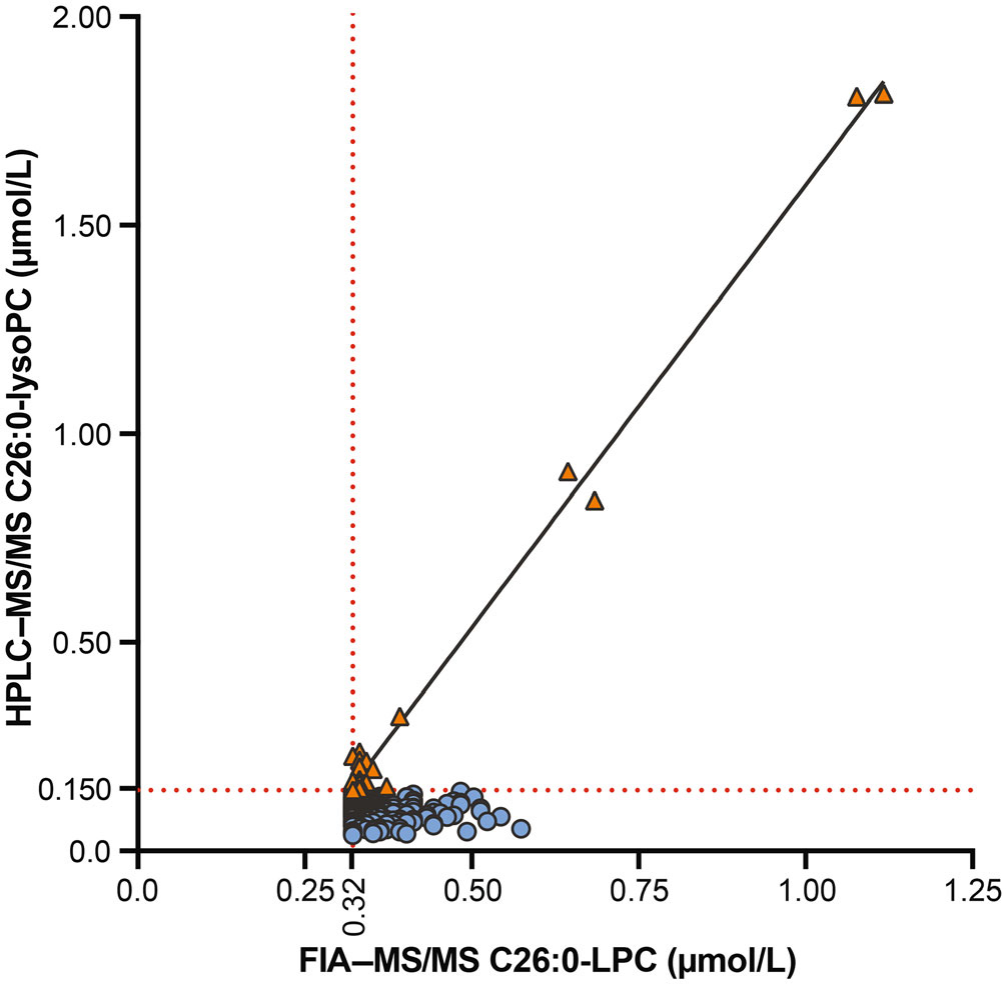
Correlation of C26:0‐LPC with FIA–MS/MS and HPLC–MS/MS. Correlation plot showing the correlation between the “putative” C26:0‐LPC (+isobaric interferent(s)) concentration measured in Tier 1 with FIA–MS/MS (X‐axis) and the (accurate) C26:0‐LPC concentration measured in Tier 3 with HPLC–MS/MS (Y‐axis). Blue circles are Tier 1 positive, but Tier 3 negative samples and orange triangles are both Tier 1 and Tier 3 positive samples. The dashed lines represent the cut‐offs for Tier 1 (≥0.32 mol/L) and Tier 3 (≥0.150 mol/L), respectively.

An as of yet unidentified isobaric component interferes with C26:0‐LPC analysis using the FIA–MS/MS method. This means that the concentration of C26:0‐LPC measured in Tier 1 does not reflect the actual concentration, but represents the sum of C26:0‐LPC + the isobaric interferent(s). The accurate C26:0‐LPC concentration is determined in Tier 3, which consists of a much more selective and sensitive HPLC–MS/MS analysis. Figure 2 shows a graphical presentation of C26:0‐LPC concentrations determined with FIA–MS/MS compared with HPLC–MS/MS during the ALD pilot (*n* = 249). The orange triangles represent the 20 dried blood spots that were positive both with FIA–MS/MS and HPLC–MS/MS. The blue circles represent the 229 dried blood spots that were positive with FIA–MS/MS (Tier 1), but negative with HPLC–MS/MS (Tier 3). Depending on the Tier 1 cut‐off chosen, the percentage of FIA–MS/MS false‐positives is above 90% (these samples are negative when analyzed by HPLC–MS/MS). Furthermore, for the 20 screen positive samples with both methods (orange triangles), there is a strong positive correlation (Pearson's *r* (19) = 0.997, *p* < 0.0001) between the C26:0‐LPC concentration measured with FIA–MS/MS (Tier 1) and the C26:0‐LPC concentration measured with HPLC–MS/MS (Tier 3). However, the reverse is not the case: the 229 samples that were negative with HPLC–MS/MS (blue circles) showed a large variation in C26:0‐LPC concentrations measured with FIA–MS/MS (Pearson's *r*[228] = 0.064, *p* = 0.335). Of note, a large number of Tier 3 negative dried blood spots had a Tier 1 C26:0‐LPC concentration that was higher than the Tier 1 C26:0‐LPC concentration of most Tier 3 positive samples. This indicates that: (1) it is likely that there is large variation in the concentration of the isobaric interferent(s), and (2) an ALD screening based purely on FIA–MS/MS would not be reliable, because the contribution of the isobaric interferent(s) to the “putative” C26:0‐LPC concentration in FIA–MS/MS is unclear and likely highly variable. In order to adhere to the recommendation of the Dutch Health Council to only screen boys for ALD, the algorithm has been designed in such a way that newborns who are screen‐positive with FIA–MS/MS, are actually screened with HPLC–MS/MS for ALD after it is determined that the newborn is a boy.

### 3.6 Covariate analysis

After completion of the pilot, and with permission of the relevant committee of the DVP (RIVM), we obtained anonymized information regarding gestational age, sex, birth weight and age at heel prick blood sampling for analysis of a possible effect of these covariates on dried blood spot C26:0‐LPC concentrations measured by FIA–MS/MS (Tier 1). After exclusion of incomplete data sets and repeat screening, data of 70 401 newborns were used for covariate analysis.

Within Europe, the Netherlands has the highest domestic birth rate (16.3% of all births in 2020)^37^ and if the child is born in the hospital, mother and child are, in general, sent home several hours after birth. As a result, 63 375 (90%) heel prick samples were collected at home, by a trained employee of the Youth Health Care. The mean age at which heel prick blood was sampled was 5 days. The majority (67 453 or 95.8%) of heel pricks was performed between 72 and 168 h after birth and 2008 (2.9%) within the next 24 h.

**FIGURE 3:**
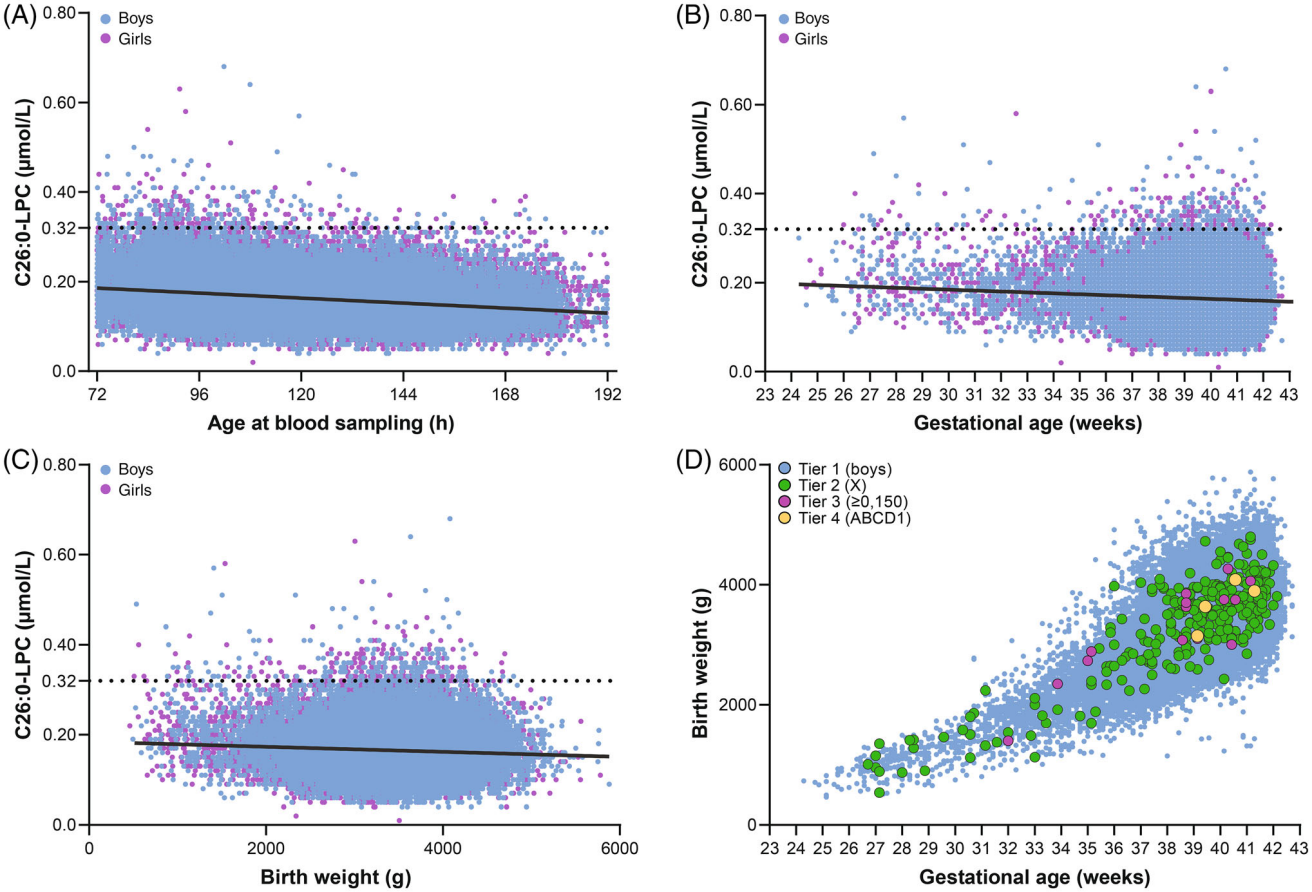
Effect of age at blood sampling, gestational age and birth weight on C26:0‐LPC. (A–C) Correlation plots showing the correlation between age of the newborn at heel prick blood sampling (A), gestational age at birth (B), birth weight (C) and C26:0‐LPC concentrations by FIA–MS/MS. Blue dots indicate males and pink dots indicate females. (D) Correlation plot showing the correlation between gestational age at birth and birth weight for all boys in Tier 1 (blue), 249 boys in Tier 2 (green), 20 screen‐positive boys in Tier 3 (pink) and the four ALD newborns (yellow).

We investigated whether the age of the newborn at heel prick blood sampling affects C26:0‐LPC concentrations and whether age‐adjusted cut‐offs are needed. Figure 3A shows that between 72 and 192 h of age there is a significant, but only small correlation between age at sampling and C26:0‐LPC levels in Tier 1 (Pearson's *r*[69,460] = −0.237, *p* < 0.001). For samples taken between 72 and 96 h after birth the mean C26:0‐LPC concentration was 0.18 ± 0.05 μmol/L (range 0.04–0.63; *n* = 18 348) and samples collected between 168 and 192 h after birth the mean was 0.15 ± 0.04 μmol/L (range 0.04–0.39; *n* = 2008) (Table 3). These data show that there is no need for age‐adjusted C26:0‐LPC cut‐offs, at least not during the first week of life.

**TABLE 3:** Effect of age at blood sampling on C26:0‐LPC (μmol/L) concentrations in Tier 1 (FIA–MS/MS)

Age	72–96 h	96–120 h	120–144 h	144–168 h	168–192 h
*N*	18 348	21 365	17 208	10 532	2008
C26:0‐LPC (mean) (μmol/L)	0.18	0.17	0.16	0.15	0.15
SD	0.05	0.05	0.05	0.04	0.04
Min	0.04	0.02	0.04	0.04	0.04
Max	0.63	0.68	0.46	0.39	0.39
97th percentile	0.29	0.27	0.25	0.25	0.25

Next, we determined whether sex affects C26:0‐LPC levels. Comparison of C26:0‐LPC concentrations in Tier 1 between girls and boys showed no effect of sex. For girls, the mean C26:0‐LPC concentration was 0.17 ± 0.05 μmol/L (range 0.01–0.63; *n* = 34 236) and for boys it was 0.16 ± 0.05 μmol/L (0.05–1.11; *n* = 36 165). Hence, boys cannot be identified based on C26:0‐LPC levels.

Furthermore, we were interested in whether gestational age at birth affects C26:0‐LPC concentrations. Mean gestational age at birth was 39 + 4 weeks (median 39 + 6 weeks; range 24 + 2–43 + 1 weeks). A total of 3892 children (5.5%) was born preterm (<37 weeks of gestation). Of these, 488 (0.7%) were born very‐preterm (<32 weeks of gestation). Comparison of C26:0‐LPC concentrations in children born preterm with those in children born full term showed a significant, but minimal effect of gestational age (Pearson's *r*[70,399] = −0.059, *p* < 0.001) (Table 4 and Figure 3B). These data demonstrate that there is no need to re‐screen a (very) preterm newborn for ALD at term age.

**TABLE 4:** Effect of gestational age on C26:0‐LPC (μmol/L) concentrations in Tier 1 (FIA–MS/MS)

Gestational age	Very preterm	Preterm	Full term
*N*	488	3404	66 508
C26:0‐LPC (mean) (μmol/L)	0.21	0.18	0.16
SD	0.07	0.05	0.05
Min	0.06	0.02	0.01
Max	0.57	0.58	0.68
97th percentile	0.35	0.29	0.26

Finally, we investigated whether birth weight affects C26:0‐LPC levels (Figure 3C). Mean birth weight was 3449 ± 557 grams (median 3480 grams; range 460–5880 grams). When birth weight is plotted against C26:0‐LPC, there is a significant, but minimal effect of birth weight on C26:0‐LPC concentrations (Pearson's *r*[70,385] = −0.058, *p* < 0.001).

The combined results show that the four newborns with ALD identified in the SCAN study were born full term with a normal birth weight (Figure 3D).

## 4 DISCUSSION

The SCAN study is the first sex‐specific newborn screening implemented in a real‐life setting. Here, we present the results of this 1‐year pilot carried out in four Dutch provinces. The recommendation made by the Health Council of the Netherlands to only screen males for ALD and not identify other conditions associated with elevated C26:0‐LPC concentrations posed a challenge. Prior to the start of the pilot, we developed and validated the X‐counter.^31^ The X‐counter enables the selection of dried bloodspots derived from male newborns, but also from newborns with Turner syndrome (45,X), for further analysis of C26:0‐LPC by HPLC–MS/MS.^31^ Importantly, the identification of a girl with both Turner syndrome and ALD is warranted, because of the same risk of developing adrenal insufficiency and/or cerebral ALD as a boy with ALD. On the other hand, the X‐counter will prevent screening for ALD of a boy with Klinefelter syndrome (47XXY), who will have the same very low risk of developing adrenal insufficiency and/or cerebral ALD as a girl with ALD. Therefore, ALD screening based on the number of X‐chromosomes is in line with the recommendation of the Dutch Health Council to only identify newborns at risk of developing adrenal insufficiency and/or cerebral ALD.

To ensure a high enough number of dried blood spots to test the screening algorithm, parents of boys and girls were asked to participate in the SCAN study. Of the 72 379 newborns that were eligible for ALD screening, parents of 1171 (1.62%) children declined participation. Parents of a girl declined participation twice as often (2.19%) as parents of a boy (1.08%). A likely explanation could be that screening for ALD is sex‐specific and therefore parents of a girl may have decided that there is no advantage in participating in the SCAN study. Overall, participation in the SCAN study was 98.4% for all eligible newborns and 98.9% for boys, which is very close to the overall newborn screening participation rate (99.3% in 2020) in the Netherlands.^38^

The identification of a variant in *ABCD1* results in referral to the pediatric neurologist. In case a known pathogenic variant is identified, only confirmatory testing of this finding is needed before the newborn enters the follow‐up program. Identification of a new variant (VUS) on the other hand causes diagnostic uncertainty. Repeat biochemical plasma VLCFA or C26:0‐LPC analysis, and/or functional testing of VLCFA metabolism in fibroblasts may be helpful in such cases,^36^ but some uncertainty may still remain. For example, diagnostic uncertainty remains when VLCFA or C26:0‐LPC concentrations are above the upper level of the reference range, but below the lower level of the established ALD disease range, which has been determined by C26:0‐LPC measurements in a large series of samples obtained from confirmed ALD patients.^34^

Over 1000 unique pathogenic variants have been registered in the *ABCD1* Variant Database (www.adrenoleukodystrophy.info),^6^ of which 520 (50.4%) affect a single amino acid residue (missense variants). Of these, 237 (47%) are classified as pathogenic, 142 (27%) as likely pathogenic, 41 (8%) as benign, but 98 (19%) are classified as a VUS. Interestingly, 97 out of 98 VUS have been reported in the last 3 years. In fact, all were identified in newborn screening, often with borderline elevated C26:0‐LPC concentrations (determined by HPLC–MS/MS) and in all cases without a family history of ALD.^22^, ^23^, ^24^, ^25^, ^26^, ^27^ A functional test in skin fibroblasts may help to classify a VUS as likely benign or likely pathogenic, but it requires taking a skin biopsy from the newborn and highly specialized diagnostic testing.^36^ Furthermore, it is currently unknown how the functional test results in skin fibroblasts translate to clinical outcome and severity of symptoms. Long‐term follow‐up of these newborns will be required to provide a definitive answer.

For parents, receiving a result of uncertain clinical significance from newborn screening can be distressing, and this negative impact may persist when uncertainty remains unresolved.^39^ At the same time, it has been well‐established that pathogenic variants in *ABCD1* have no predictive value with respect to clinical outcome. This is a complicating factor in ALD disease management.^4^, ^5^, ^6^, ^7^ Indeed, many pathogenic variants including variants that have a clearly deleterious effect on the ABCD1 protein (like large deletions, nonsense variants and frame shifts) have been associated with the entire clinical spectrum of ALD.^40^, ^41^

Currently, it is not known whether there are any (missense) variants in *ABCD1* that result in borderline elevated VLCFA or C26:0‐LPC levels, but do not cause ALD or cause only a late‐onset mild phenotype. Population screening will identify boys with borderline elevated C26:0‐LPC concentrations and a new variant in *ABCD1*. The question whether these children are at risk of developing adrenal insufficiency and/or cerebral ALD or whether these variants are exclusively associated with a late‐onset mild phenotype (which before newborn screening may not have been recognized as ALD) can only be answered with long‐term follow‐up, open collaboration and the sharing of (anonymized) information. However, the fact that since the start of ALD newborn screening a high number of new missense variants and newborns without a family history of disease have been reported, may already indicate that indeed both non‐disease‐causing variants (benign variants) and variants associated with mild and/or late‐onset clinical symptoms are being identified. For these newborns and their families, the “burden of knowledge” likely outweighs the clinical benefit.^42^ Preventing the identification of newborns with *ABCD1* variants and C26:0‐LPC concentrations outside the normal range, but well below the ALD disease range, can be resolved relatively easy by raising the cut‐off of the highly sensitive HPLC–MS/MS (confirmatory Tier 3) to a C26:0‐LPC concentration closer to the established ALD disease range. For example, in our case a decision can already be made, based on the data of Tier 3 (Table 1), to increase the Tier 3 cut‐off from 0.150 to 0.200 μmol/L. This will not jeopardize the primary objective of ALD newborn screening, that is, the identification of boys who are at risk of developing adrenal insufficiency and/or cerebral ALD. Importantly, a higher cut‐off will have two advantages: (1) it limits the number of genetic tests performed in Tier 4, and thereby (2) it reduces the chance of finding boys with a benign variant in *ABCD1*.

One of the main goals of our pilot was to test the sex‐specific algorithm in a real‐life setting. Therefore, parents of girls were asked to participate in the SCAN study and the cut‐offs for both Tier 1 (FIA–MS/MS) and Tier 3 (HPLC–MS/MS) were set rather low. That way, a relatively high number of samples would be screen‐positive in Tier 1 and therefore a sufficient number of samples could be analyzed in the subsequent tiers. We anticipate that when nationwide ALD screening starts these cut‐offs may be increased either immediately or after a run‐in phase.

We identified four boys with elevated C26:0‐LPC concentrations and a variant in *ABCD1*. In one newborn a known pathogenic variant (p.Arg617His) was identified. This variant has been reported in 58 ALD cases (*ABCD1* Variant Database)^6^ and is associated with all possible clinical outcomes. In three children likely pathogenic variants (p.Asn192Asp, p.Arg418Gln, c.1781‐1G>C) were found. Following referral, the diagnosis ALD was confirmed in all four newborns. Therefore, the positive predictive value (PPV) of the ALD pilot screening algorithm was 100% (4/4). Extended family screening is still ongoing, but none of the families reported a history of ALD. In the absence of a predictive test for clinical outcome and severity, all four newborns enrolled in a long‐term follow‐up program to enable prospective monitoring and timely therapeutic intervention. Currently, we cannot rule out that we have identified mild or late‐onset variants, or even non‐disease‐causing variants. Of note, the variant p.Arg418Gln has also been identified recently in four newborns in California and Pennsylvania.^22^, ^24^ Establishing whether a new variant is (likely) benign or (likely) pathogenic is of great importance for diagnostic counseling and clinical follow‐up. Therefore, our current research program is focused on the identification of specific (lipid) biomarkers in blood that correlate with disease severity, with the aim to provide insight into the pathogenicity of new variants and to improve care of newborns as well as counseling of their families.

Analysis of a possible effect of covariates on C26:0‐LPC levels measured by FIA–MS/MS demonstrated that gestational age and birth weight have only a minimal effect on C26:0‐LPC concentrations, which is a new and important finding. This means that there is no need to re‐screen a preterm newborn for ALD at term age. Also, comparison of C26:0‐LPC concentrations in girls and boys showed no effect of sex. Hence, boys cannot be identified based on C26:0‐LPC levels. Furthermore, our data shows that C26:0‐LPC is minimally affected by age of heel prick blood sampling, at least during the first week of life, rendering age‐adjusted C26:0‐LPC cut‐offs unnecessary.

Based on the results of screening of 71 208 newborns for ALD, the birth prevalence of ALD in this population was 1 boy with ALD in 18 000 newborns screened, or 1 boy with ALD in 9000 boys screened. However, the number of screened newborns in the Netherlands is still relatively small and therefore the true prevalence of ALD in the Netherlands is still an estimation.

## 5 CONCLUSION

The SCAN study has shown that it is feasible to employ a boys‐only screening algorithm that identifies males with ALD without identifying unsolicited findings, based on elevated C26:0‐LPC levels, the presence of one X‐chromosome and a variant in *ABCD1* in heel prick dried bloodspots. This approach will be of interest to countries that are considering ALD newborn screening but are reluctant to identify girls with ALD because for girls there is no direct health benefit. Furthermore, our analysis of the effect of gestational age, sex, birth weight and age at heel prick blood sampling on C26:0‐LPC concentrations demonstrates that these covariates have a minimal effect on C26:0‐LPC. Finally, the identification of four male newborns with increased C26:0‐LPC concentrations and (likely) pathogenic *ABCD1* variants will allow prospective monitoring and timely therapeutic intervention, thereby preventing irreversible damage and saving lives. It is expected that nationwide ALD newborn screening in the Netherlands will start in 2023.

## AUTHOR CONTRIBUTIONS

Marc Engelen and Stephan Kemp conceived the project. Eugènie Dekkers, Marc Engelen, and Stephan Kemp obtained funding. Samantha L. van der Beek, Inge M. E. Dijkstra, Mariëlle Alders, Jet Bliek, Anita Boelen, Merel S. Ebberink, Sacha Ferdinandusse, Susan M. I. Goorden, Annemieke C. Heijboer, Mandy Jansen, Yorrick R. J. Jaspers, Ingrid Metgod, and Frédéric M. Vaz performed and/or supervised the laboratory analyses. Monique Albersen and Stephan Kemp analyzed the data. Samantha L. van der Beek, Rinse W. Barendsen, Jet Bliek, Anita Boelen, Gajja S. Salomons, Frédéric M. Vaz, Susan M. I. Goorden, Rendelien K. Verschoof‐Puite, Wouter F. Visser, Eugènie Dekkers, Marc Engelen, and Stephan Kemp organized the SCAN study, referral, and follow‐up. Monique Albersen, Marc Engelen, and Stephan Kemp wrote the manuscript.

## FUNDING INFORMATION

The SCAN study is funded by the Netherlands Organization for Health, Research and Development (ZonMw), project number 543002004 to Stephan Kemp.

## CONFLICT OF INTEREST

Monique Albersen, Samantha van der Beek, Inge Dijkstra, Mariëlle Alders, Rinse Barendsen, Jet Bliek, Anita Boelen, Merel Ebberink, Sacha Ferdinandusse, Susan Goorden, Annemieke Heijboer, Mandy Jansen, Yorrick Jaspers, Ingrid Metgod, Gajja Salomons, Rendelien Verschoof‐Puite, Wouter Visser, and Eugènie Dekkers declare that they have no conflict of interest. Frédéric Vaz has received consulting fees from Scenic Biotech outside the submitted work. Marc Engelen has received unrestricted research grants from Minoryx, SwanBio Therapeutics, Bluebird Bio, and AutoBahn Therapeutics separate from the submitted work; has received consulting fees from Minoryx, Swanbio Therapeutics, Bluebird Bio, AutoBahn Therapeutics, and Poxel for scientific advising outside the submitted work; participates in advisory board the United Leukodystrophy Foundation (unpaid). Stephan Kemp has received unrestricted research grant support from Bluebird Bio and Swanbio Therapeutics separate from the submitted work; has received consulting fees from Poxel and Swanbio Therapeutics for scientific advising outside the submitted work; participates in advisory boards for ALD Connect (unpaid), the European Leukodystrophy Association (unpaid), Alex, The Leukodystrophy Charity (unpaid), and the United Leukodystrophy Foundation (unpaid).

## ETHICS STATEMENT

This article does not contain any studies with human subjects performed by any of the authors. The study protocol was reviewed by the Institutional Review Board from the Amsterdam UMC (METC W18_433 # 19.017), and deemed exempt, because the Medical Research Involving Human Subjects Act (WMO) does not apply.

## PATIENT CONSENT STATEMENT

Informed consent for participation in the SCAN study was requested. If parents decided not to participate this was marked on the Guthrie card. These cards were excluded from analyses.

## DOCUMENTATION OF APPROVAL FROM THE INSTITUTIONAL COMMITTEE FOR CARE AND USE OF LABORATORY ANIMALS

This article does not contain any studies with animal subjects performed by any of the authors.

## Data Availability

The data that support the findings of this study are available on request from the corresponding author. These data are not publicly available due to privacy or ethical restrictions.
